# Molecular Diet Analysis of Two African Free-Tailed Bats (Molossidae) Using High Throughput Sequencing

**DOI:** 10.1371/journal.pone.0021441

**Published:** 2011-06-22

**Authors:** Kristine Bohmann, Ara Monadjem, Christina Lehmkuhl Noer, Morten Rasmussen, Matt R. K. Zeale, Elizabeth Clare, Gareth Jones, Eske Willerslev, M. Thomas P. Gilbert

**Affiliations:** 1 Centre for GeoGenetics, Natural History Museum of Denmark, Copenhagen, Denmark; 2 All Out Africa Research Unit, Department of Biological Sciences, University of Swaziland, Kwaluseni, Swaziland; 3 School of Biological Sciences, University of Bristol, Bristol, United Kingdom; Institut de Biologia Evolutiva - Universitat Pompeu Fabra, Spain

## Abstract

Given the diversity of prey consumed by insectivorous bats, it is difficult to discern the composition of their diet using morphological or conventional PCR-based analyses of their faeces. We demonstrate the use of a powerful alternate tool, the use of the Roche FLX sequencing platform to deep-sequence uniquely 5′ tagged insect-generic barcode cytochrome *c* oxidase I (COI) fragments, that were PCR amplified from faecal pellets of two free-tailed bat species *Chaerephon pumilus* and *Mops condylurus* (family: Molossidae). Although the analyses were challenged by the paucity of southern African insect COI sequences in the GenBank and BOLD databases, similarity to existing collections allowed the preliminary identification of 25 prey families from six orders of insects within the diet of *C. pumilus*, and 24 families from seven orders within the diet of *M. condylurus*. Insects identified to families within the orders Lepidoptera and Diptera were widely present among the faecal samples analysed. The two families that were observed most frequently were Noctuidae and Nymphalidae (Lepidoptera). Species-level analysis of the data was accomplished using novel bioinformatics techniques for the identification of molecular operational taxonomic units (MOTU). Based on these analyses, our data provide little evidence of resource partitioning between sympatric *M. condylurus* and *C. pumilus* in the Simunye region of Swaziland at the time of year when the samples were collected, although as more complete databases against which to compare the sequences are generated this may have to be re-evaluated.

## Introduction

Molecular techniques have become a practical tool for investigating the diets of vertebrates [1], [2], particularly those that are difficult to observe such as nocturnal bats [3]–[6]. Conventional microscope-based faecal analyses are problematic in insectivorous bats as they masticate their insect prey thoroughly, and often do not swallow the hard parts by which the insects can be more readily identified [7] Although conventionally limited to PCR amplification of target prey fragments [3], [4] or via whole faecal extraction coupled with molecular cloning [6] the increasing availability of the next generation sequencing techniques (the so-called ‘High-Throughput DNA Sequencers’), offers the potential to transform molecular diet analyses, making it cost effective on a large scale [8]–[12]. For example, Roche's FLX sequencer, in its current incarnation running Titanium sequencing chemistry, can sequence up to 500 megabases (Mb) of sequence in a single run from as many as 1 million sequences generated in parallel. In order to further increase the power and economy of the method, uniquely tagged primers [13] can be used to amplify each specific DNA template source, thereby enabling the parallel sequencing of amplicons from all faecal samples whilst tracking the origin of prey sequences [11], [12]. Although a wide range of insect samples have sequence data available on databases such as the Barcode of Life Data Systems (BOLD – see http://www.barcodinglife.com) for the identification of recovered DNA fragments [3], bio-informatic analyses are also available that allow species level analysis in the absence of suitable reference libraries [4] making the techniques applicable in all situations.

In this study, we analysed the diet of two sympatric free-tailed bats in Swaziland, *Chaerephon pumilus* and *Mops condylurus* (Molossidae), by exploiting the power of high-throughput sequencing coupled to novel bioinformatic analyses. The two species are relatively closely related, belonging to the subfamily Molossinae, and having high aspect ratios and wing loadings, hence are adapted for flight in open habitats. Furthermore they roost together in the study area, and have been observed to feed together over local sugarcane fields, an area with known high concentrations of insects (Ara Monadjem, unpublished observations). Previous studies based on microscopic examination of faecal pellets have shown that both these bats feed on a wide variety of insect orders [7], [14], [15]. Both species use echolocation calls with similar peak frequencies (*C. pumilus* 25–40 kHz, *M. condylurus* 26–35 kHz [15]) to orientate and locate their prey in flight [7], thus suggesting that they may not partition prey resources via echolocation [16], [17] This hypothesis is contradicted by a previous microscopic faecal analysis of the two species in Uganda, Kenya and Malawi [18], which concluded that morphological differentiation was responsible for perceived resource partitioning between the two species and enabled them to survive sympatrically [18]. However, given their taxonomic relatedness, similar behaviour and hunting strategies, we hypothesised that resource partitioning may actually be minimal between these two species, particularly in areas of high insect diversity and abundance, and that more powerful molecular based analyses may demonstrate this.

Given the above, we have used high-throughput sequencing and bioinformatic techniques to investigate the diet of these species in order to test our hypothesis with regards to resource partitioning, and to provide a preliminary identification of insects comprising the two bat species' diet. Since the two bat species roost together and faecal collections were made non-invasively by collecting them from under roosts, primers amplifying a 152 bp fragment of the bat mitochondrial 16S rRNA gene were used to assign a species of origin to each pellet. Generic insect primers with unique tags were used to amplify mini barcode cytochrome *c* oxidase I (COI) fragments (157 bp) of prey insect DNA extracted from faecal pellets [6], and Roche GS-FLX sequencing was used for rapid and parallel sequencing of PCR amplicons from all faecal samples. We discuss in detail, the potential and limitations experienced when using these techniques.

## Materials and Methods

Bat material studied included faecal pellets and wing biopsies. The faecal pellets were taken non-invasively, thus require no ethical approval. Wing biopsies were obtained in strict accordance with the guidelines published in *Guidelines of the American Society of Mammalogists for the use of Wild Mammals in Research* [19]), on recommendation and under approval from both the Natural History Museum of Denmark (University of Copenhagen), and the Department of Biology (University of Swaziland). The method used (wing membrane biopsy) is not classed by American Society of Mammalogists as detrimental to the bats, due to the rapid healing of the wound and lack of evidence of any negative side effects (e.g. [20]). Bat sampling was furthermore undertaken under Skov- og Naturstyrelsen/CITES sampling permit DK003, as granted to the Natural History Museum of Denmark.

### Study site

The study area was located in and around Simunye, Tambankhulu and Ngomane villages in the north-eastern Swaziland lowveld, adjacent to Hlane Royal National Park, Mbuluzi Game Reserve and Mlawula Nature Reserve (between 26°06′S to 26°13′S and 31°48′E to 31°55′E).

The land use of the study site is primarily either sugarcane plantations or protected areas. The adjacent Hlane Royal National Park, Mbuluzi Game Reserve and Mlawula Nature Reserve are a contiguous network of protected areas covering almost 500 km^2^ [21]. The dominant vegetation in this network is classified as lowveld microphyllous (*Acacia*) savanna, but patches of riparian forest occur along rivers and major drainage lines [22].

### Sample collection

#### Collection of wing biopsies

Wing biopsies were collected from adult bats to use as a source of reference DNA, against which the faecal pellets could be assigned to the source species. Biopsies were sampled from 20 adult *C. pumilus* and *M. condylurus* individuals, captured as they departed from the day roost in the roof of a house near Simunye village. Wing biopsies were taken using a biopsy punch sterilised with 96% ethanol and burned under a flame. The wing of the bat was spread out on a solid, flat surface covered with a clean sheet of paper, and a biopsy was taken where blood vessels were not present and was transferred to a 2 ml tube in 96% ethanol. The wing biopsies were stored at ambient temperature in the field (<3 months), and at −18°C in the lab.

#### Collection of faece

Faecal samples were collected in the austral autumn, between April and May 2009, from four molossid roosts (all in roofs of houses) in which both bat species co-habit. The location of the roosts was as follows (1) Magistrate's Court House (−26.18604; 31.90538), (2) Tambankhulu (−26.10584; 31.92034), (3) Ngomane House (−26.194666, 31.81234), (4) Simunye (−26.2071; 31.92429). The shortest and longest distances between the four sites were 2.94 km (site 1 and 4) and 14.61 km (site 2 and 3), respectively. Thus given this proximity and the similarity of the landscape at each site, we hypothesise that any observed difference in diet between the species is unlikely to be explained through access to different species of insects. Each roost was sampled 3–5 times, and on each occasion 11–20 pellets were collected overnight (120 faecal samples in total). Faeces were collected on boards, raised above the ground, which were placed horizontally underneath the exit hole of the bat roosts. To minimise the risk of contamination, the boards were covered with new cling film on each night of sampling. Pellets were stored separately in 2 ml tubes following the ‘two-step’ storage procedure described by Nsubuga et al. [23]. Immediately after collection, 96% ethanol was added to each tube until it covered the pellets and the tubes were mixed by inversion. After 24–36 hours the ethanol was carefully poured off and silica beads (Silica gel type III, Sigma-Aldrich ® S7625-500 G) were added to desiccate the samples.

#### Collection of insects

The Swaziland Sugar Association (SSA) provided samples of insects that had been collected in the sugarcane fields, to be used in insect primer optimisation trials.

### DNA extraction, amplification and sequencing

#### DNA extraction of faeces

DNA was extracted from approximately half of each pellet (ca. 0.002–0.005 g). The pellets were cut in half using a scalpel that had previously been flame-sterilised in 96% ethanol. The extractions were carried out using the QIAamp DNA Stool Mini Kit (Handbook 07/2007) (Qiagen, Valencia, CA) according to the manufacturer's instructions with modification following Zeale et al. [6] where in step 1, 0.002–0.005 g of faecal material was used, and in step 5, only half an InhibitEX tablet was added to each sample.

DNA was extracted from all 120 faecal samples, with a maximum of 36 samples extracted at a single time and with four extraction blanks included to check for crossover contamination. Extracted DNA was stored at −18°C prior to subsequent PCR analyses.

#### DNA extraction of wing biopsies

Wing biopsies were extracted using a DNeasy Blood and Tissue kit (Qiagen) protocol for purification of total DNA from animal tissues. The extractions were carried out according to the manufacturer's instructions with the modification that 100 µl of Buffer AE was used in step 7. Extracted DNA was kept at −18°C prior to subsequent PCR analyses.

#### DNA extraction of insects

1 to 2 legs of each insect were cut off using a flame-ethanol sterilised scalpel. The legs were transferred to a 1.5 ml tube and 200 µl of a digestion buffer following Gilbert et al. [24] was added. Each sample was vortexed and incubated at 56°C overnight with agitation. DNA from insect samples was subsequently purified from the buffer using the QIAquick PCR purification kit (Qiagen), following the manufacturer's protocol with the following modifications: In step (1) 1000 µl Buffer PB was added. Step (2) was not carried out. In step (4) 600 µl of sample was added and each column was centrifuged for 1 minute at 6,000 x g. Flow-through was discarded. The remaining sample was added and the column was centrifuged for 1 minute at 6,000 x g. In step (6) 750 µl Buffer PE was added to each column and the column was centrifuged for 1 minute at 10,000 x g. In step (9) 50 µl Elution buffer EB was added to each QIAquick membrane and the column was centrifuged for 1 minute at 6,000 x g. The extracted DNA was kept at −18°C prior to subsequent PCR analyses.

### DNA amplification and sequencing

#### Faecal source identification

The primer design program Primer3 [25] as implemented in the Geneious analytical package (www.geneious.com) was used to design a single set of primers that could be used to identify the species of origin for the collected faecal pellets. As a single primer set was required that could identify both bat species, they were designed to target a relatively conserved region of the mitochondrial DNA 16S rRNA gene. Primers were developed using the criteria: (*i*) the primer sequences should be conserved between *C. pumilus* and *M. condylurus.* Sequences for the two species were found in Genbank: *C. pumilus*: AY495454.1 and *M. condylurus*: AY495456.1, (*ii*) the primers should not be able to bind to human DNA (sequence found in Genbank) or insect DNA, (*iii*) the primers should amplify a fragment of around 100–200 bp length, to maximise their chance of working on the degraded DNA that is likely to be present in faeces [26]. In addition, conventional primer design rules were followed to help ensure amplification specificity and success. From these criteria, the 16S bat primer sequences were designed that yield an expected amplicon size of 152 bp (102 bp excluding primers):

Forward, bat 16S F: ACGAGAAGACCCTATGGAGCTTT (annealing temperature 55°C)

Reverse, bat 16S R: AGTCTAGGCTTAAAATCACTCGGAAGT (annealing temperature 55°C)

Wing biopsies from *C. pumilus* and *M. condylurus* were sequenced in order to confirm that the primers produced sequence that was distinguishable between the two species. During faecal PCR reactions, DNA from wing biopsies was included as a positive control.

#### PCR protocol for 16S primers on faeces and wing biopsies

PCRs were performed in 25 µl PCR reactions using the Amplitaq Gold enzyme system (Roche, Basel, Switzerland). Each reaction contained 1 µl DNA from faeces or wing biopsies, 1x PCR Gold Buffer, 2.5 mM MgCl_2_ solution, 200 nM each dNTP, 0.1 µl AmpliTaq Gold, and 400 nm of each primer. Cycling was performed using a DNAEngine Peltier Thermal Cycler (Bio-Rad Laboratories, Hercules, CA) with the following cycle program: Initial denaturation at 95°C for 5 minutes followed by 50 cycles of 95°C for 30 seconds, 56°C for 30 seconds and 72°C for 30 seconds, followed by a final extension at 72°C for 7 minutes and 4°C forever. 5 µl of the PCR products were visualised through running at 100 V for 40 minutes on 2% agarose gels stained using ethidium bromide. For size comparison PCR products were run against a 50 bp ladder.

Positive PCR-products were purified using the MSB(R) Spin PCRapace (Invitek, Westberg, Germany) protocol according to the manufacturer's instructions with the following modifications: In step 2, 15 µl elution buffer was added to each sample and each sample was incubated for 3 minutes. Sanger sequencing of the products (both directions) was carried out by the commercial facility offered by Beckman Coulter Genomics (Takeley, UK).

#### Insect barcode (COI) primers

Insect DNA was amplified from the faeces using insect generic COI primers ZBJ-ArtF1c and ZBJ-ArtR2c that yield an amplicon of ca. 157 bp – located within and at the 5′ end of the standard 658 bp COI barcode region – across a wide range of insect orders [6]. Prior to experimental use in this study the efficiency of the primers was verified on a range of potential local insect species confirming widespread ability to amplify DNA from a variety of common bat prey groups. These included *Sesamia calamistis* Hampson (Lepidoptera: Noctuidae), *Eldana saccharina* Walker 1865 (Lepidoptera: Pyralidae), *Busseola fusca* Fuller (Lepidoptera: Noctuidae), *Chilo sacchariphagus* Bojer 1856 (Lepidoptera: Crambidae), *Mythimna phaea* Hampson 1902 (Lepidoptera: Noctuidae), *Schizonycha affinis* Boheman 1857 (Coleoptera: Scarabaeidae), *Heteronychus licas* Klug 1835 (Coleoptera: Scarabaeidae), *Anomala ustulata* Arrow 1899 (Coleoptera: Scarabaeidae) and *Astenopholis dasypus* Burmeister 1855 (Coleoptera: Scarabaeidae).

#### PCR protocol for insect barcode (COI) primers on DNA from insects

PCR reactions were performed in 25 µl PCR reactions as above. Cycling was performed using a DNAEngine Peltier Thermal Cycler (Bio-Rad Laboratories) with the following cycle program: Initial denaturation at 95°C for 5 minutes followed by 50 cycles of 95°C for 30 seconds, 52°C for 30 seconds and 72°C for 30 seconds, followed by a final extension of 7 minutes at 72°C and 4°C forever. PCR products were visualised and purified as above, with Sanger sequencing of the products (both directions) undertaken by the commercial facility offered by Macrogen (Seoul, South Korea).

#### Fusion insect barcode (COI) primers on DNA from faeces

To enable deep sequencing of the insect DNA present in the bat faeces using a Roche FLX sequencer, the insect barcoding primers were modified into ‘fusion primers’, following the manufacturer's guidelines. Fusion primers consist of the original target primer, extended at the 5′ end by 19–27 bp of sequence (primer dependent). For the modified ZBJ-ArtF1c primer, the 5′ 19 bp consisted of a specific primer binding site, used in the emulsion-based clonal amplification (emPCR) required by the FLX platform, and the subsequent 8 bp consisted of unique DNA barcodes (tags), which can be used to segregate different sequences generated by the FLX, into original source DNA extracts. In this study, the ZBJ-ArtF1c primer was modified using 30 different tags. The reverse primer (ZBJ-ArtR2c) was untagged, and contained only the reverse FLX-specific 19 bp extension at the 5′ end. To enable the generation of PCR product from all 120 DNA extracts, using unique primer combinations, the total extracts were subdivided into five groups, which were kept separate at all subsequent analytical steps. Five PCR reactions were performed according to the above-mentioned protocol for the barcode primers. Every tube in each batch had its own distinct ZBJ-ArtF1c-30 primer. 5 µl of the PCR products were visualised through running at 100 V for 40 minutes on 2% agarose gels, stained using ethidium bromide. For size comparison PCR products were run against a 50 bp ladder. If primer-dimers were present, gel cuts were performed in order to avoid sequencing the primer-dimers. Purification of the gel cuts were performed using the Eppendorf Perfectprep ® Gel Cleanup kit according to the manufacturer's instructions. Finally, all samples were double purified to remove all residual small DNA fragments using the Agencourt AMPure XP ® protocol (96 well format).

#### FLX sequencing of amplicons

The fusion amplicons were deep-sequenced using a Roche FLX following the manufacturer's guidelines for fusion primers. Prior to emPCR and sequencing, PCR products from each subgroup were pooled into a single group, at an equimolar ratio. This was achieved by first quantifying the number of PCR amplicons within each PCR product using real time PCR (qPCR) incorporating a DNA standard of known concentration, following Meyer et al. [27], and using the Roche emPCR primers for amplicon sequencing. All samples were diluted 1:1000 in TE-Tween buffer (0.05% Tween 20 in 1x TE buffer) following Meyer et al. [27]. Real time PCR was performed using a LightCycler (R) 480 II (Roche), and the enzyme Amplitaq Gold (Roche), in 25 µl PCR volumes. Each reaction contained 1 µl DNA, 1x buffer, 2.5 mM MgCl_2_, 100 nM each dNTP, 0.1 µl Amplitaq Gold, 400 nM of each primer, and 1 µl SYBR Green/Rox mix (Invitrogen, Carlsbad, CA).

### Data analyses

The pellets were assigned to a bat species through comparison of the sequenced 16S sequences from the pellets against the reference sequences generated from wing biopsy material. Sufficient nucleotide differences exist between the two species over the amplified region to unambiguously assign pellets to the species (14 SNPs and 2 deletions over the 102 bp amplified).

### Analyses of insect prey content in the faecal pellets

In order to provide preliminary identifications of what insect families were present among the prey, a customised Perl script was used to compare the obtained sequences against the NCBI nt nucleotide sequence database, using the software BLAST (ncbi.nlm.nih.gov), followed by visualisation of the results using MEGANv3.8 [28] (http://ab.inf.uni-tuebingen.de/), here all taxonomic matches of each read are evaluated and the lowest common taxonomic level is assigned. In order to exclude spurious PCR amplicons that were not derived from the target insect DNA, or incompletely sequenced amplicons from the analysis, only sequences of ca. 157 bp (exact length depended on presence of indels in sequence arising due to sequencing error) were used for these analyses. Different files of BLAST comparisons were assembled for the MEGAN analyses, (1) files containing prey sequences for each pellet, and (2) files containing prey sequences for each bat species. Within these BLAST-files, we initially eliminated all prey sequences that only appeared once in the dataset, as a conservative approach undertaken in order to prevent overestimation of sample genetic diversity arising due to sequencing errors. Subsequently, to reduce computation time during downstream analyses, the remaining sequences were collapsed so that each unique sequence only appeared once.

In order to assign a taxon to each sequence in the BLAST-files, MEGAN processed the BLAST data of each sequence to determine all the hits. Hits below the threshold for the bit-score of hits (min. score, standard setting  = 35.0) were discarded. Furthermore, hits were discarded due to the threshold for the maximum percentage (top percentage, standard setting  = 10) by which the score of a hit may fall below the best score achieved for the sequence. After collecting the hits that exceeded the thresholds, MEGAN found the lowest node that encompassed all these hits using the LCA-assignment algorithm (LCA = Lowest Common Ancestor) to assign sequences to taxa. Minimum support was set to 1, implying that only one sequence had to be assigned to a taxon in order for the taxa to appear in the resulting cladogram. Other settings used were standard settings. In this way, the resulting cladograms provided an overview of the taxonomical distribution and abundance of different prey sequences.

When analysing data in MEGAN, if a sequence aligned specifically only to a single taxon it was assigned to that taxon. The less specifically a sequence hit taxa, the higher up in the taxonomy it was placed. Due to a general paucity of Swazi (or South African) insects in the Barcode of Life Data System (www.boldsystems.org), it was not possible to assign a low taxonomic level following Clare et al. [3], therefore, data analyses were carried out on family and order-level using Linnean taxonomy and at the species level using bio-informatics methods (below).

#### General diet of Chaerephon pumilus and Mops condylurus

For each pellet, the number and diversity of individual insect sequences attributed to families and orders were registered. For both bat species, these data were used to calculate the percent frequency of occurrence for each insect family among the pellets (the percentage of pellets that a given insect family occurred in). Prey accumulation curves at the family level were calculated using EstimateS v7.5 [29]. The recovered data were statistically analysed to provide further insights into the distribution of the insect families among the pellets and between bat species.

#### Species level analysis – using molecular operational taxonomic units

In the absence of species level identifications we employed the methods of Clare et al. [4] to estimate the number of prey species consumed by each bat species using the program jMOTU (http://www.nematodes.org/bioinformatics/jMOTU/index.shtml, Anisah Goorah, Martin Jones and Mark Blaxter) which groups sequences by a user-defined boundary of similarity. jMOTU identifies molecular operational taxonomic units in groups of sequences which can be used as a proxy for alpha-level taxonomy (see [30]) and has been successfully applied to unidentifiable bat prey in molecular analysis [4].

We aligned all sequences using a reference and calculated MOTU at a 2.5% cut-off threshold for both species (see Clare et al. [4] for a discussion of parameter choice). Using assigned MOTU for each haplotype as a proxy for prey species identification we recorded the frequency of detection for each MOTU. To determine whether the two bat species consumed a similar number of MOTU we calculated the mean number of MOTU consumed by each individual bat. To estimate dietary richness (niche size) we constructed MOTU accumulation curves and we calculated the diversity of species in each diet using the Simpson and Shannon diversity indices which include measures of both the number of species and the evenness of their representation. for each bat species using EstimateS v7.5 [29] with 50 random resamplings of the data. To explore niche overlap, and the degree of resource partitioning between these two species, we isolated only those MOTU which were detected in more than one faecal sample (n = 49) and determined the proportion which were consumed multiple times by only one predator species versus those consumed by both species.

## Results

The expected 16s bat and CO1 fragments from prey insects were PCR amplified and sequenced from 89 of the 120 (74.2%) analysed pellets. In the remaining 31 pellets we were unable to amplify prey DNA. Of the 89 pellets, 30 pellets were from *M. condylurus* and 59 were from *C. pumilus*.

### General diet of Chaerephon pumilus and Mops condylurus

A total of 35,808 sequence copies of ca. 157 bp were recovered from the pellets of both bat species. After removal of sequences only appearing in single copy (conservatively assumed to be sequencing errors) and collapsing the sequences, a total of 1,646 unique haplotypes were recovered. Comparisons with the NCBI nt database (www.ncbi.nlm.nih.gov) allowed preliminary identification of 90.95% of these unique sequences to the class Insecta or to a lower taxonomic level within the insects (see discussion on limitations of identifications). The remaining sequences either showed sequence similarity to non-target organisms (such as bacteria, fungi, algae, dogs, humans, mice, bats), likely derived from either laboratory contaminants or erroneous binding of the primers to non-insect DNA within the pellets, or were unidentifiable because either (i) nothing similar was represented in the NCBI database, or (ii) they were possibly chimeric sequences [31].

**Figure 1:**
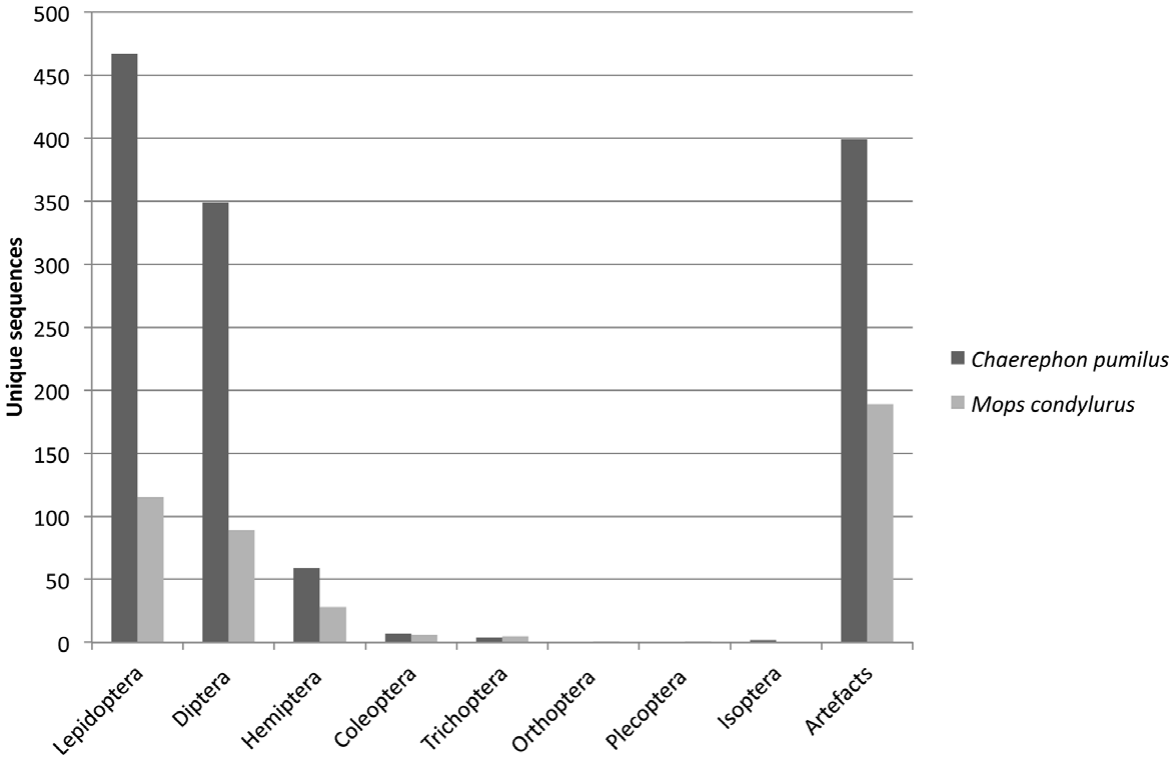
The overall distribution of unique sequences determined in pellets from *Chaerephon pumilus* (n = 59), and *Mops condylurus* (n = 30). The column ‘artefacts’ refers to sequences that could not be assigned to Insecta.

The overall distribution of unique sequences found in the pellets of *C. pumilus* and *M. condylurus* showed that for both bat species, most unique sequences are found within the insect orders Lepidoptera and Diptera (Fig. 1).

#### Chaerephon pumilus

A total of 1,287 unique haplotypes were recovered from the 59 pellets of *C. pumilus*. Comparisons with the NCBI database allowed the identification of 90.4% (1,163 unique sequences) of these to the class Insecta and 27.6% (355 unique sequences) to a specific family of insects. A total of six insect orders containing 25 families were found within the prey of *C. pumilus* (Table 1). The mean number of unique insect haplotypes per pellet was 23.3 (SD ±29.35, range 1–222).

**Table 1:** Frequency of occurrence of all insect families obtained from 59 pellets from *Chaerephon pumilus*.

Order	Family	Common name/description	Number of pellets containing given family	Pellet occurrence frequency (%)
**COLEOPTERA (Beetles)**	Dytiscidae	Predaceous diving beetles	1	1.7
	Hydrophilidae	Water scavenger beetle	1	1.7
**Total coleopteran families**			**1**	**1.7**
**DIPTERA (Flies)**	Chironomidae	Non-biting midges	1	1.7
	Culicidae	Mosquitoes	16	27.1
	Drosophilidae	Pomace flies	1	1.7
	Muscidae	House flies and kin	1	1.7
	Sciomyzidae	Marsh flies	1	1.7
	Stratiomyidae	Soldier flies	1	1.7
	Tephritidae	Fruit flies and kin	6	10.2
**Total dipteran families**			**24**	**40.7**
**HEMIPTERA (True bugs)**	Aphididae	Aphids	1	1.7
	Lygaeidae	Chinch bugs and seed bugs	6	10.2
	Miridae	Plant bugs, leaf bugs, grass bugs	1	1.7
	Pentatomidae	Stink bugs	6	10.2
**Total hemipteran families**			**11**	**18.6**
**ISOPTERA (Termites)**	Termitidae	Termites	2	3.4
**Total isopteran families**			**2**	**3.4**
**LEPIDOPTERA (Moths and butterflies)**				
	Crambidae**	Grass moths	6	10.2
	Geometridae**	Geometer moths	4	6.8
	Hesperiidae	A family of skipper butterflies	1	1.7
	Noctuidae**	Owlet moths	15	25.4
	Nymphalidae*	Brush-footed butterflies	18	30.5
	Oecophoridae	A family of moths	1	1.7
	Pyralidae**	Snout moths	1	1.7
	Saturniidae	Saturniids	1	1.7
	Sphingidae*	Hawk moths, sphinx moths, hornworms	1	1.7
	Tortricidae	Tortrix moths	1	1.7
**Total lepidopteran families**			**31**	**52.5**
**TRICHOPTERA (Caddisflies)**	Hydropsychidae	Net-spinning caddisflies	1	1.7
**Total trichopteran families**			**1**	**1.7**

*Families that include some tympanate species.** Families entirely comprised of tympanate species.

**Figure 2:**
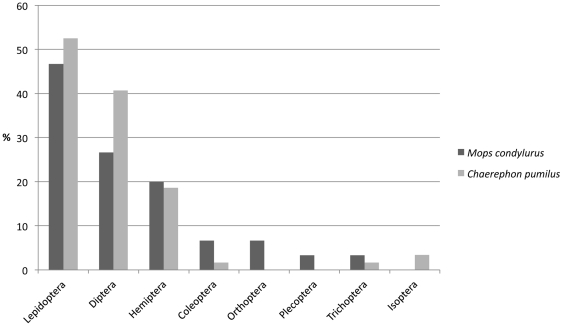
Frequency of occurrence (percent of pellets) of insect orders (with assigned families) in the diet of *Chaerephon pumilus* (n = 59) and *Mops condylurus* (n = 30).

Insects from the orders Lepidoptera and Diptera had the highest frequency of occurrence with lepidopterans found in 52.5% and dipterans in 40.7% of the pellets (Table 1; Fig. 2). Within Lepidoptera, the families Nymphalidae, Noctuidae and Crambidae had the highest frequency of occurrence. Within Diptera, the family Culicidae had the highest frequency of occurrence (Table 1).

#### Mops condylurus

A total of 434 unique sequences were found in the 30 pellets of *M. condylurus*. Comparisons with the NCBI database allowed the identification of 93.6% (406 unique sequences) of these to the class Insecta and 25.6% (111 unique sequences) to a specific family of insects. A total of seven insect orders containing 24 families were found within the prey of *M. condylurus*. The mean number of unique insect haplotypes per pellet was 15.5 (SD ±10.35, range 3–46).

Insects from the orders Lepidoptera and Diptera had the highest frequency of occurrence with lepidopteran insects found in 46.7% and dipteran insects in 26.7% of the pellets (Table 2; Fig. 2). As with *C. pumilus,* within Lepidoptera, the families Nymphalidae, Noctuidae and Geometridae had the highest frequency of occurrence, and within Diptera, the families Drosophilidae and Muscidae had the highest frequency of occurrence (Table 2).

**Table 2:** Frequency of occurrence of all insect families obtained from 30 pellets from *Mops condylurus*.

Order	Family	Common name/description	Number of pellets containing given family	Pellet occurrence frequency (%)
**COLEOPTERA (Beetles)**	Cerambycidae	Longhorn beetles	1	3.3
	Dytiscidae	Predaceous diving beetles	1	3.3
	Scarabaeidae	Scarab beetles	1	3.3
**Total coleopteran families**			**2**	**6.7**
**DIPTERA (Flies)**	Calliphoridae	Carrion flies	1	3.3
	Drosophilidae	Pomace flies	2	6.7
	Muscidae	House flies and kin	2	6.7
	Sarcophagidae	Flesh flies	1	3.3
	Sciomyzidae	Marsh flies	1	3.3
	Simuliidae	Black flies	1	3.3
**Total dipteran families**			**8**	**26.7**
**HEMIPTERA (True bugs)**	Lachnidae	Aphids	1	3.3
	Lygaeidae	Chinch bugs and seed bugs	2	6.7
	Pentatomidae	Stink bugs	2	6.7
	Scutelleridae	Shield-backed bugs	1	3.3
**Total hemipteran families**			**6**	**20**
**LEPIDOPTERA (Moths and butterflies)**	Coleophoridae	Family of moths	1	3.3
	Geometridae**	Geometer moths	3	10
	Hesperiidae	Skippers	1	3.3
	Noctuidae**	Owlet moths	7	23.3
	Nymphalidae*	Brush-footed butterflies	5	16.7
	Pyralidae**	Snout moths	1	3.3
	Sphingidae*	Hawk moths, sphinx moths, hornworms	1	3.3
	Tortricidae	Tortrix moths	2	6.7
**Total lepidopteran families**			**14**	**46.7**
**ORTHOPTERA (Grasshoppers, crickets etc.)**	Gryllidae**	Crickets	2	6.7
**Total orthopteran families**			**2**	**6.7**
**PLECOPTERA (Stoneflies)**	Nemouridae	Spring stoneflies	1	3.3
**Total plecopteran families**			**1**	**3.3**
TRICHOPTERA (Caddisflies)	Hydropsychidae	Net-spinning caddisflies	1	3.3
**Total trichopteran families**			**1**	**3.3**

*Families that include some tympanate species.** Families entirely comprised of tympanate species.

#### Prey accumulation curves

**Figure 3:**
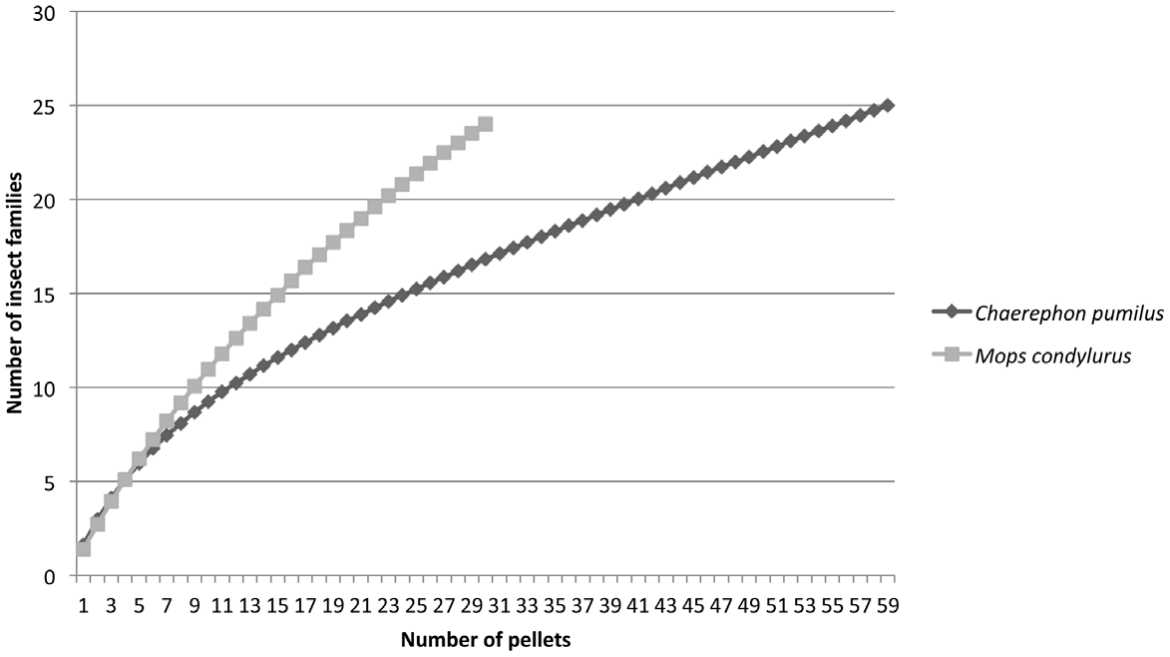
Prey accumulation curve (Colwell 2005) for prey insect families identified in the faeces of *Chaerephon pumilus* and *Mops condylurus*. Number of pellets corresponds to sampling intensity.

Accumulation curves of the prey families in the diet of *C. pumilus* and *M. condylurus* (Fig. 3a & b) did not appear to reach plateaus. This suggests that the 25 and 24 identified prey families, respectively, do not represent the complete diet of these two bat species.

#### Comparisons of the diet of Chaerephon pumilus and Mops condylurus

**Figure 4:**
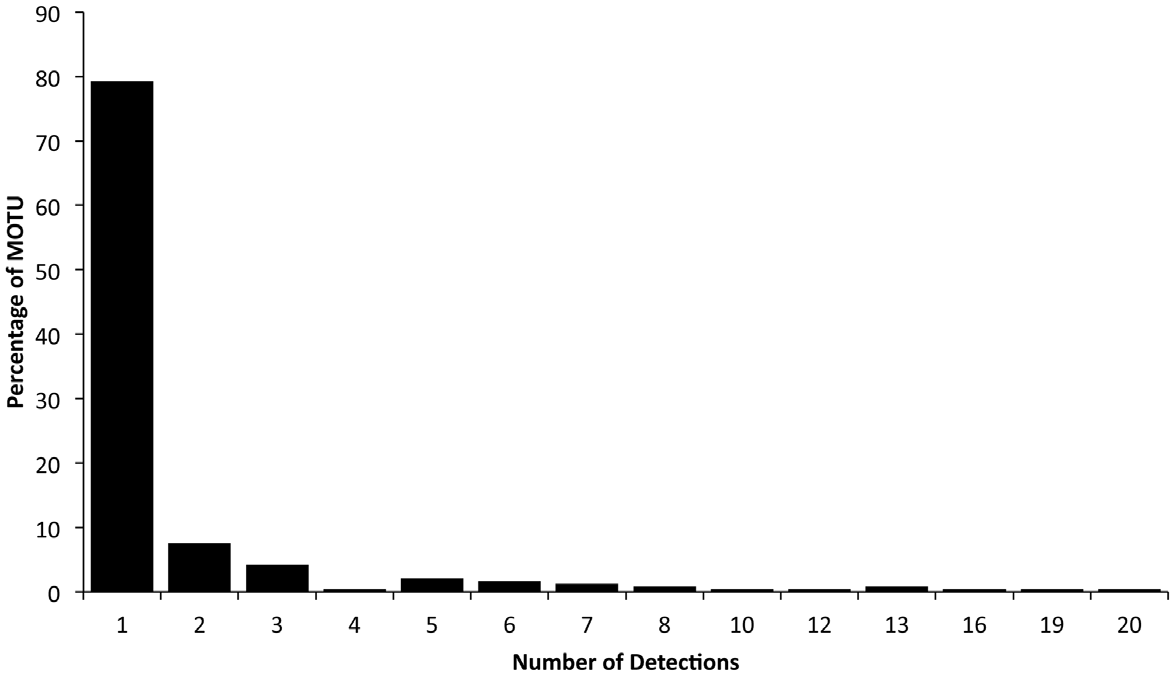
Distribution of MOTUs among pellets.

**Figure 5:**
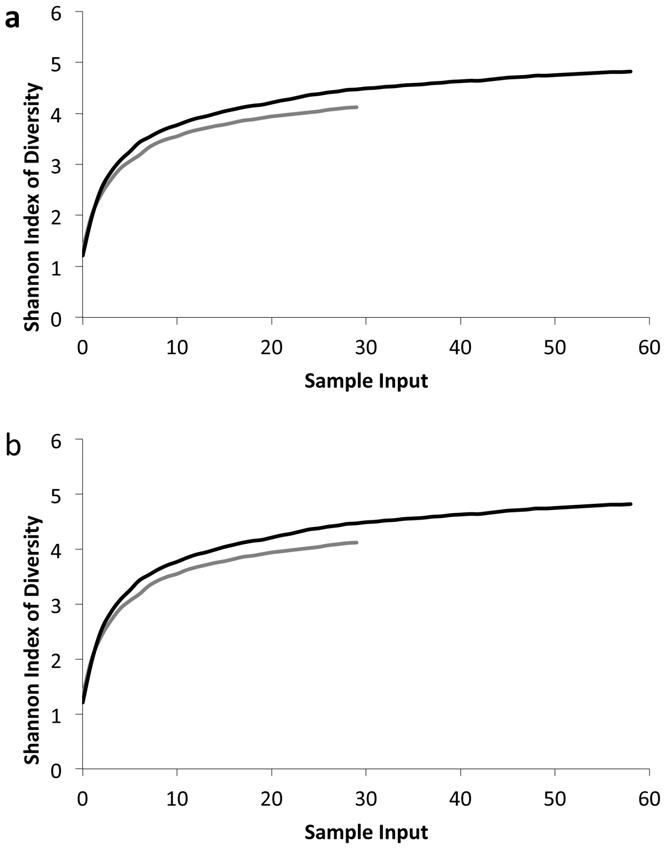
Diversity indices based on resampling of MOTUs. (Fig. 5a) Shannon Index, (Fig. 5b) Simpson Index.

A total of 1874 haplotypes were successfully aligned to reference sequences and subsequently collapsed into 236 unique MOTU using a 2.5% threshold in the program jMOTU. The majority of MOTU (∼79%) were detected in only one guano pellet with the most common MOTU detected in 20 different pellets (Fig. 4). The average number of MOTU detected per faecal sample did not differ statistically between species. MOTU accumulation curves and Simpson and Shannon diversity indices for each species based on resampling of MOTU results suggest that *C. pumilus* consumes a wider range of species than *M. condylurus* (Fig. 5a &b). This finding was consistent when a random subsample of 30 *C. pumilis* pellets was analysed (data not shown), indicating that the larger size if the *C. pumilis* dataset was not the cause of this observation. Among MOTU detected multiple times, more than 50% of cases of multiple consumption of MOTU were between species, and the null hypothesis that there is no difference in MOTU consumption between species cannot be rejected. Given that the incidence of overlapping consumption exceeds that of multiple consumption within species, there is no obvious case for resource partitioning among these data.

## Discussion

The principal aim of the study was to characterise molecularly the dietary diversity of *C. pumilus* and *M. condylurus* in order to investigate whether there is any evidence of resource partitioning of their prey. In addition, we aimed to validate whether the chosen method may be of use in this context for bats. Since few other dietary studies have used uniquely tagged primers and high-throughput FLX sequencing to sequence numerous different faecal DNA samples in parallel [8]–[12], we discuss in Discussion S1 the opportunities and limitations experienced when using this technique.

While our analysis suggests that the diet of *C. pumilus* is broader than that of *M. condylurus* for common prey items (i.e. those detected more than once) there was substantial dietary overlap - more prey species were detected multiple times among heterospecific bats than among conspecific bats. Thus we cannot make a strong case for resource partitioning between these species – at least during the time of year when the samples were taken. In contrast to our study, Happold & Happold [18], using microscopic faecal analysis, showed that sympatric *C. pumilus* and *M. condylurus* fed on different insect orders. They concluded that morphological differentiation enabled resource partitioning and enabled these two bat species to survive sympatrically [18]. The difference may be due to the species-level resolution of our data. Clare et al. [4] noted strong differences in the classification of “generalist” and “specialist” for two sympatric bat species when data were analysed at the species versus family level. In addition, the diversity and richness of insects in the study area may exceed the predation pressure exerted by these two species, thus reducing competition and the requirement for resource partitioning. It is not possible to extrapolate from sequence/haplotype frequency to frequency of insects eaten (see Discussion S1), thus our study was neither able to quantify the number of insects eaten by individual bats nor the availability of insect prey, so our data are limited to quantification of estimates based on presence/absence of MOTU between pellets. Thus, it was also impossible to determine the proportion of insects with hard (e.g. beetles) or soft (e.g. moths) integuments eaten by these two species.

Some additional caveats need to be borne in mind when interpreting the results of this study. First, faeces were analysed from four different sites over a relatively short period of time. Since these bats have previously been shown to be opportunistic feeders that adapt their diet to the different kinds of insects available during the year [18], our data cannot reflect the full spectrum of prey taken by these bats. This is supported by our prey accumulation curves that did not reach asymptotes for either bat species (Fig. 3a & b). Second, the mean numbers of unique insect sequences per pellet were 15.5 and 23.3 for *M. condylurus* and *C. pumilus*, respectively. This may well be an underestimate due to the incomplete prey reference database, the removal of singletons, and the relatively short barcoding fragments targeted [32], perhaps resulting in some insect species having the same sequence over this fragment. It is interesting to note that these different measures of haplotypes/pellet in each species did not translate into a different number of MOTU estimated per pellet. While this demonstrates that haplotype frequency is unlikely to accurately represent abundance of insect species (see Discussion S1), it does suggest that the insects consumed by *C. pumilus* may be more genetically diverse (possessed more haplotypes / species) than those consumed by *M. condylurus*, but that the behaviour of the bats in terms of insect captures and digestion chemistry is similar, i.e. the same number of insect taxa are likely to be captured and passed in each pellet by each bat species, an important consideration when designing similar studies.

Although molecular methodologies have been gaining popularity as a tool for dietary analysis, they have been used in only a few studies for bats [3]–[6]. In all cases, these methods have been applied to species inhabiting areas where substantial taxonomically curated genetic reference collections are available, and can be used to identify recovered sequences, and provide species level resolution to the data. Lack of prey reference collections have represented a severe restriction to the widespread application of these analysis though, ironically, it is in areas with little known faunas (eg. tropical areas) where molecular analyses are most needed. To counter this, Clare et al. [4] adopted a bio-informatics approach from the molecular taxonomic community, to estimate the number of species that could not be identified in conjunction with genetic reference collections. In our case, the study area has little or no reference collection and we rely exclusively on the MOTU approach for species-level analysis in the absence of Linnaean taxonomy. There are several caveats to this, for instance, threshold approaches to species delimitation assumes that all species are defined by similar levels of genetic variability which will be increasingly problematic as the target group gets more and more diverse. While this is a limitation, there are ranges of diversity that are biologically relevant (see Clare et al [4] for a discussion of this) and can be used to estimate taxonomic diversity. In particular, our use of 2.5% as a cutoff was chosen as a midpoint in the diversity reported for many large insect orders (e.g. [33]) and permits us to answer questions about niche size and niche overlap at the species level even in this relatively unknown biological fauna. As such, this method represents a substantial advance for these analyses making them applicable in any ecosystem. It should also be noted, that using a cutoff for a clustering algorithm like MOTU is not the same as the sequence similarity approach for species identification used by Clare et al. [3], Zeale et al. [6] and Clare et al. [4]. The identification of a sequences to species level [3], [4] is more complicated and has been done using sequence similarity measures alone [6], and a combined similarity and phylogenetic approach [3], and is of course most accurate when species level matches are perfect.

One intriguing observation from the data is that the most abundant prey families identified in the pellets of *C. pumilus* and *M. condylurus* are tympanate (Table 1 and 2), and hence have defences that may help them detect and avoid predation [34] by *C. pumilus* and *M. condylurus*, which echolocate within the frequency range of best hearing of most tympanate insects [15]. If the sequence assignment here is accurate, then the data indicates that *C. pumilus* and *M. condylurus* are sometimes able to bypass the defence strategy of tympanate insects. Clare et al. [3] suggested that the ability of *Lasiurus borealis* to feed on a variety of tympanate species, even though its echolocation calls should be audible to insect prey, may be due to differential defensive behaviour of flying insects [35], making them vulnerable to predation at night around artificial light sources. However, this is not likely to be the case in our study, as artificial lights were scarce. An alternative explanation might be that these bats are foraging in areas of high prey density, where the relative advantage of appearing less conspicuous to tympanate prey would be reduced [36]. This corresponds well with the findings of a recent radio-tracking study which showed that *C. pumilus* and *M. condylurus* preferred to forage over sugarcane fields (Christina Lehmkuhl Noer, unpublished data), over which insects appeared to be abundant.

At a more general level, the results of this study support the conclusions of Clare et al. [3] and Zeale et al. [6] that DNA from a range of insect prey regularly survive the journey through the bats' digestive system, and that PCR amplification using barcoding primers that amplified short multi-copy COI fragments could be used to generate detailed datasets of prey content. Bearing in mind that short fragments amplified from faeces were targeted and the reference database was incomplete for the study region, prey insect orders and families were assigned to a relatively high proportion of sequences though these should be considered as preliminary (see Clare et al. [4] and see Discussion S1 for these limitations). A valid question is whether the use of the conventional barcoding primers that amplify a longer (648 bp) fragment of COI might have enhanced the ability to identify the prey [4], [37]. Given the current sequencing capability of the FLX platform (ca. 400–500 bp read lengths), with anticipated length increases in the near future of up to 800 bp reads, such analyses will soon be possible. Despite relatively high success rates of amplification of long fragments of DNA from fresh bat faeces [3], more difficulty has been encountered when the faeces is not preserved immediately [4], suggesting a rapid process of DNA degradation. It will be important to investigate whether any taxonomic specific DNA degradation biases exist, through comparison of results generated using both long and short amplifications, thus caution should be taken when relying on longer PCR amplifications and there may be a trade-off between length for species-level identification [4] and length for efficient recovery of amplicons (see Discussion S1). In general however, we believe that the approach adopted here is broadly applicable to study the diet of other bats and perhaps other generalist insectivores - especially in studies where non-invasively collected faecal samples are preferred, where faecal samples from sympatric insectivores are analysed simultaneously and in studies conducted in areas for which there is good coverage in the prey reference database. Furthermore, these methods are particularly well suited for large-scale analyses, since they offer the possibility to analyse many samples in the same FLX sequencing run and to automate the sequence analysis by implementing bioinformatic tools.

## Supporting Information

Discussion S1 Opportunities and Limitations of FLX Sequencing in Dietary Analyses.
(DOCX)
