# Supplementary material for: Molecular Diet Analysis of Two African Free-Tailed Bats (Molossidae) Using High Throughput Sequencing
Source: PLoS One. 2011 Jun 22;6(6):e21441. doi: 10.1371/journal.pone.0021441 (PMC3120876; doi:10.1371/journal.pone.0021441)
Supplement: Discussion S1 — Opportunities and Limitations of FLX Sequencing in Dietary Analyses. (DOCX) [file pone.0021441.s001.docx]

**Molecular diet analysis of two African free-tailed bats (Molossidae) using high throughput sequencing**

Kristine Bohmann, Ara Monadjem, Christina Lehmkuhl Noer, Morten Rasmussen, Matt R. K. Zeale, Elizabeth Clare, Gareth Jones^3^, Eske Willerslev, M. Thomas P. Gilbert

**Supplemental Discussion**

**OPPORTUNITIES AND LIMITATIONS OF FLX SEQUENCING IN DIETARY ANALYSES**

Overall, the use of high-throughput FLX sequencing, instead of traditional cloning and sequencing of clones, increases the number of samples it is possible to analyse within the same time and cost frame [S1-S3], and increases the identification of diversity within pellets [S4]. Since the use of FLX sequencing increases the information obtained, it can be assumed, that the prey accumulation curves came closer to reaching a plateau than would have been possible if traditional cloning and sequencing had been performed within the same time and cost frame.

**Qualitative dietary analyses**

Although the approach used in this study ultimately yields a ‘copy number’ of each unique DNA sequence, these numbers cannot currently be interpreted as quantitative as DNA molecule frequency may become biased during PCR due to a number of factors, including preferential primer annealing to certain templates, and stochasticity of the PCR amplification process, as well as factors discussed below [S2, S5]. Therefore, when interpreting such sequencing results it is important to keep in mind that the techniques used in this study alone cannot assess the quantity of different insect families eaten within a sample (guano pellet). Thus, it provided an estimate of the frequency of occurrence of insect families among the faeces, but not an estimate of the volume of insects eaten from those families. The implications of this, is that although a great sequence diversity of, for example, insects within the order Lepidoptera was observed in faeces from both bat species, the volume of these insects eaten might have been small. On the other hand, families from which few unique sequences were identified in the pellets might have been eaten in great numbers by the bats.

As discussed below, several biological and technical factors made it unlikely that the proportion of mitochondrial DNA copies from the recovered insect families would reflect the proportions of insect biomass ingested by the bats.

It has previously been shown that DNA proportions of food items in faeces may provide a biased reflection of the true proportions of prey eaten, as DNA survival during digestion may vary between prey species (e.g. [S3, S7]), and as the copy number of mitochondrial DNA may vary between tissues, prey species, reproductive states etc., creating additional natural sources of amplification bias (e.g. [S3, S5, S6, S7]).

Apart from these biological issues, there are technical issues that can affect how well prey DNA in the faeces are amplified. DNA from all food items will be amplified in equal amounts if completely conserved primer binding sites are targeted. Unfortunately, it is hard to identify primers that are completely conserved, and will amplify fragments that are short enough to be suitable for faecal DNA amplification while being informative, and well-represented in GenBank [S7]. If conserved primers are not used some prey might be preferentially amplified over others [eg. S7].

With these limitations in mind, prey-DNA extracted from faeces will accurately reflect the amounts of prey eaten if DNA copy number is similar between both prey species and tissues consumed, if the DNA from different prey survives similarly through the digestive process, and if prey-DNA is amplified with primers that are appropriately conserved between prey species [S2]. If these criteria are met, FLX-sequencing might be used to quantify food items in the diet [S2].

**Secondary predation and scavenging**

It is possible, that the obtained insect sequences might not derive from insects that the bats caught and killed themselves. Scavenging and secondary predation are ways for prey to appear in faeces without the predator having killed it itself, [S8-S10]. Scavenging is when a predator consumes a dead animal that were not killed to be eaten by the predator. Since *C. pumilus* and *M. condylurus* catch their prey in flight, it is unlikely, that the identified prey insects could have been scavenged. Secondary predation is when the primary predator eats a target prey, and is then itself eaten by a secondary predator. When the diet components of the second predator is analysed it may seem like the second predator ate a prey item directly while it was truly a case of secondary predation. This problem arises because PCR amplification of prey DNA cannot discriminate between food items that were eaten directly by the predator or originated in the diet due to secondary predation [S8, S9]. In a DNA-based study on an aphid-spider-carabid system, secondary predation was found to be a significant potential source of error in molecular diet analyses [S9]. The problems of scavenging and secondary predation have not yet been investigated in vertebrates, but can also be assumed to be a source of error in these systems [S10]. Therefore, there is a small chance that some insects were identified in the pellets because they were recently eaten by insects that were eaten by the bats. Among the insect families identified in the pellets, the most likely case of secondary predation would be that caddisfly larvae (Trichoptera) were eaten by diving beetles (Coleoptera: Dytiscidae), which were then preyed upon by the bats (Philip Francis Thomsen, pers. comm.). Since these two insect families were not frequently encountered in the bat pellets and, secondary predation was considered of minor importance in this study. It should be noted that the importance of these as sources of error will depend on the goals of the study. If an analysis of diet is of primary importance, items ingested through either process are legitimate dietary components, however if the primary goal is an analysis of predation behaviours such as predator hunting vs. prey defences, these will necessarily contaminate the analyses.

**Difficulties with assigning sequences to insect families**

Comparisons with the NCBI database and subsequent MEGAN analyses only allowed identification of 25.6% and 27.6% of the total amount of unique sequences to specific families of insects for *C. pumilus* and *M. condylurus*, respectively. In Canada, another PCR and sequence based dietary study on faeces from the eastern red bat (*Lasiurus borealis*) was able to identify 78% of the obtained sequences (109-648 bp) to species [S11]. The low proportions of sequences identified to families or lower taxonomical levels in this study, compared to the Canadian study, were caused by the relatively short size of the barcoding fragments, and the fact that few southern African insect species have been sequenced over the COI barcoding region.

Targeting short fragments of around 150 bp optimises the amount of DNA that can be amplified from the fragmented DNA present in faeces [S12, S13] while at the same time it reduces the amount of information that can be obtained when analysing the sequences [S8]. Therefore, Huson et al. [S14] investigated the importance of fragment length when using the software MEGAN (Metagenome Analysis Software) (www-ab.informatik.uni-tuebingen.de) to analyse data. They found, that since short fragments were harder for MEGAN to classify, using short fragments would result in an underestimation of the results. Furthermore, when using short fragments, MEGAN assigned many of the sequences to a higher taxonomic level than if a longer fragment had been used, and many sequences had no matches. This means, that when using MEGAN to analyse short fragments the resulting cladogram will most likely be a high level summary, there will be a relatively high amount of no matches, and there will be an underestimation of assigned sequences. But most importantly, Huson et al. [S14] found no false-positive predictions. Therefore, the diversity of the bats’ diet in this study was likely an underestimate of the true diversity but the short fragment length could be assumed to not result in false matches to taxa.

If conserved primers are used and the possible prey species are well-represented in the reference database it will be possible to identify both expected and unexpected prey in the animal's diet. This was not the case for the southern African region where the insect fauna had not been thoroughly studied systematically and molecularly as evidenced by the poorly developed Barcode of Life Data System for Swazi and South African insects: In March 2010 there were 545,389 insect specimens with barcodes in the Barcode of Life Data System ([www.boldsystems.org](http://www.boldsystems.org)). Of these, only 75 specimens came from Swaziland and 1,917 from South Africa, compared to 154,987 from Canada and 93,584 from the United States. When using MEGAN to analyse sequences that were not represented in the database, Huson et al. [S14] predicted a low rate of false-positive assignments but fairly large numbers of unspecific assignments of no matches. Therefore, the incomplete database gave an uncertainty when assigning insects to families. This could be seen in the relatively low proportion of sequences that could be assigned to insect families in this study compared to the aforementioned Canadian study [S11]. A solution to this would be to capture, identify and sequence all southern African insects to obtain a well-developed reference database. If such had been available, it would have been possible to identify prey insects down to even species-level as was possible in the aforementioned Canadian study [S11]. In this Canadian study, 127 species of insects were identified in the guano from 56 individuals of eastern red bat (*Lasiurus borealis*). This was accomplished by comparing the obtained sequences to the approximately 127,000 reference sequences derived from North American arthropods that were present in the Barcode of Life Data System in February 2008. Maybe if the sequences obtained in this study are compared against the NCBI database in a few years time, when there might be a better sequence-cover of the southern African region, a higher proportion of sequences assigned to insect families might be obtained and sequences might be assigned to lower taxonomical levels.

**Sequencing errors**

Compared to the traditional Sanger-based methods one of the drawbacks of FLX sequencing, is the relatively higher sequencing error rate. This arises due to difficulties when determining base pair counts in homopolymer stretches (identical bases in succession, as in the 3’ end of the region amplified in this study), and nucleotide misincorporation (e.g. [S14, S15]). It can be assumed, that the independent nature of the sequence generation process will mean that these kinds of sequencing errors affecting different molecules differently i.e. stochastically. Thus, any modified molecule can be assumed to only be observed at the level of few copies [S5]. Therefore, in this study, a conservative approach was adopted to try to correct for sequencing errors, through the exclusion of all sequences that only appeared in a single copy from the data analyses and alignment of sequences to a reference for MOTU analysis. The aim of this was to get rid of most sequencing errors, and as a consequence it likely lead to an underestimation of the diversity.

**Supplemental References**

S1. Binladen J, Gilbert MTP, Bollback JP, Panitz F, Bendixen C et al. (2007) The use of coded PCR primers enables high-throughput sequencing of multiple homolog amplification products by 454 parallel sequencing. PLoS ONE 2:e1-9.

S2. Deagle BE, Kirkwood R, Jarman SN (2009) Analysis of Australian fur seal diet by pyrosequencing prey DNA in faeces. Mol Ecol 18:2022–2038.

S3. Pegard A, Miquel C, Valentini A, Coissac E, Bouvier F et al. (2009) Universal DNA-based methods for assessing the diet of grazing livestock and wildlife from faeces. J Agr Food Chem 57:5700-5706.

S4. Rasmussen M, Cummings LS, Gilbert MTP, Bryant V, Smith C et al. (2009) Response to comment by Goldberg et al. on “DNA from pre-Clovis human coprolites in Oregon, North America. Science, 325:148-d.

S5. Valentini A, Miquel C, Nawaz MA, Bellemain E, Coissac E et al. (2009) New perspectives in diet analysis based on DNA barcoding and parallel pyrosequencing: the trnL approach. Mol Ecol Res 9: 51-60.

S6. Deagle BE, Tollit DJ, Jarman SN, Hindell MA, Trites AW et al. (2005) Molecular scatology as a tool to study diet: analysis of prey DNA in scats from captive Steller sea lions. Mol Ecol 14:1831-1842.

S7. Deagle BE, Gales NJ, Evans K, Jarman SN, Robinson S et al. (2007) Studying seabird diet through genetic analysis of faeces: a case study on macaroni penguins (*Eudyptes chrysolophus*). PLoS ONE 9:e831.

S8. Symondson WOC (2002) Molecular identification of prey in predator diets. Mol Ecol 11:627-641.

S9. Sheppard SK, Bell J, Sunderland KD, Fenlon J, Skervin D et al. (2005). Detection of secondary predation by PCR analyses of the gut contents of invertebrate generalist predators. Mol Ecol 14: 4461-4468.

S10. King RA, Read DS, Traugott M, Symondson WOC (2008) Molecular analysis of predation: a review of best practice for DNA-based approaches. Mol Ecol 17:947-963.

S11. Clare EL, Fraser EE, Braid HE, Fenton BM, Hebert PDN (2009) Species on the menu of a generalist predator, the eastern red bat (*Lasiurus borealis*): using a molecular approach to detect arthropod prey. Mol Ecol 18:2532-2542.

S12. Zaidi RH, Jaal Z, Hawkes NJ, Hemingway J, Symondson WOC (1999) Can multiple-copy sequences of prey DNA be detected amongst the gut contents of invertebrate predators? Mol Ecol 8: 2081-2087.

S13. Deagle BE, Eveson JP, Jarman SN (2006) Quantification of damage in DNA recovered from highly degraded samples – a case study on DNA in faeces. Front Zool 3:11.

S14. Huson DH, Auch AF, Qi J, Schuster SC (2007) MEGAN analysis of metagenomic data. Genome Res 17:377-386.

S15. Gilbert MTP, Binladen J, Miller W, Wiuf C, Willerslev E et al. (2007). Recharacterization of ancient DNA miscoding lesions: insights in the era of sequencing-by-synthesis. Nucleic Acids Res 35:1–10.
